# The depressive state of Denmark during the COVID-19 pandemic

**DOI:** 10.1017/neu.2020.15

**Published:** 2020-04-22

**Authors:** Kim Mannemar Sønderskov, Peter Thisted Dinesen, Ziggi Ivan Santini, Søren Dinesen Østergaard

**Affiliations:** 1 Department of Political Science, Aarhus University, Aarhus, Denmark; 2 Department of Political Science, University of Copenhagen, Copenhagen, Denmark; 3 The Danish National Institute of Public Health, University of Southern Denmark, Copenhagen, Denmark; 4 Department of Affective Disorders, Aarhus University Hospital – Psychiatry, Aarhus, Denmark; 5 Department of Clinical Medicine, Aarhus University, Aarhus, Denmark

**Keywords:** COVID-19, well-being, depressive disorder, major

## Introduction

The ongoing COVID-19 pandemic (World Health Organization (WHO), 2020) is likely to have negative health consequences beyond those caused by the virus per se. As outlined in the recent paper by Druss (2020), a medical field likely to experience significant consequences of the pandemic and its accompanying societal changes is that of psychiatry. Indeed, there are studies suggesting that there may be a general worsening of mental health in the populations affected by the pandemic. In a recent survey from China, 54% of the respondents rated the COVID-19 outbreak to have a moderate or severe negative psychological impact (Wang *et al.*, 2020). A similar tendency was seen in a survey conducted in the USA by the American Psychiatric Association (2020). However – in both cases – there were no prior survey data targeting the same population available to allow for a benchmark comparison. Therefore, the aim of the present study was to measure the level of psychological well-being in Denmark during the COVID-19 pandemic and to compare it to prior Danish data obtained with the same measure.

## Methods

We commissioned the survey agency ‘Epinion’ to conduct an online survey [the COVID-19 Consequences Denmark Panel Survey 2020 (CCDPS 2020)], which included the five-item WHO-5 well-being scale (Topp *et al.*, 2015) – a widely used and psychometrically valid measure of psychological well-being experienced over the past 2 weeks. The WHO-5 score ranges from 0 (minimum well-being) to 100 (maximum well-being). The survey also contained six questions regarding the experienced level of anxiety/depression over the past 2 weeks reported on a scale from 0 (not present) to 10 (present to an extreme degree). The survey was fielded from March 31 to April 6, 2020 and was completed by 2458 respondents. After weighting (applied in all analyses), the sample is representative of the population on key demographic and political variables (gender, age, education, region and party choice in the last election).

We compared two properties of the WHO-5 well-being scale from the CCDPS 2020 with those from a previous survey, namely the Danish Mental Health and Well-Being Survey 2016 (DMHWBS 2016 – see the Supplementary Material for a description) (Nielsen *et al.*, 2017), the WHO-5 mean score (two-sample *t*-test, one-sided *p*-value) and the proportion of individuals who had WHO-5 scores <50, for whom assessment for depression is recommended when the WHO-5 is used as a screening tool by general practitioners (two-sample test of proportions, one-sided *p*-value) (Topp *et al.*, 2015). Finally, the relationship between the reported symptom levels of anxiety/depression and the WHO-5 scores from the CCDPS 2020 was characterised by Spearman’s correlation coefficients. Based on the known gender differences in the prevalence of anxiety/depression, we also conducted analyses stratified by gender.

## Results

**Fig. 1.**
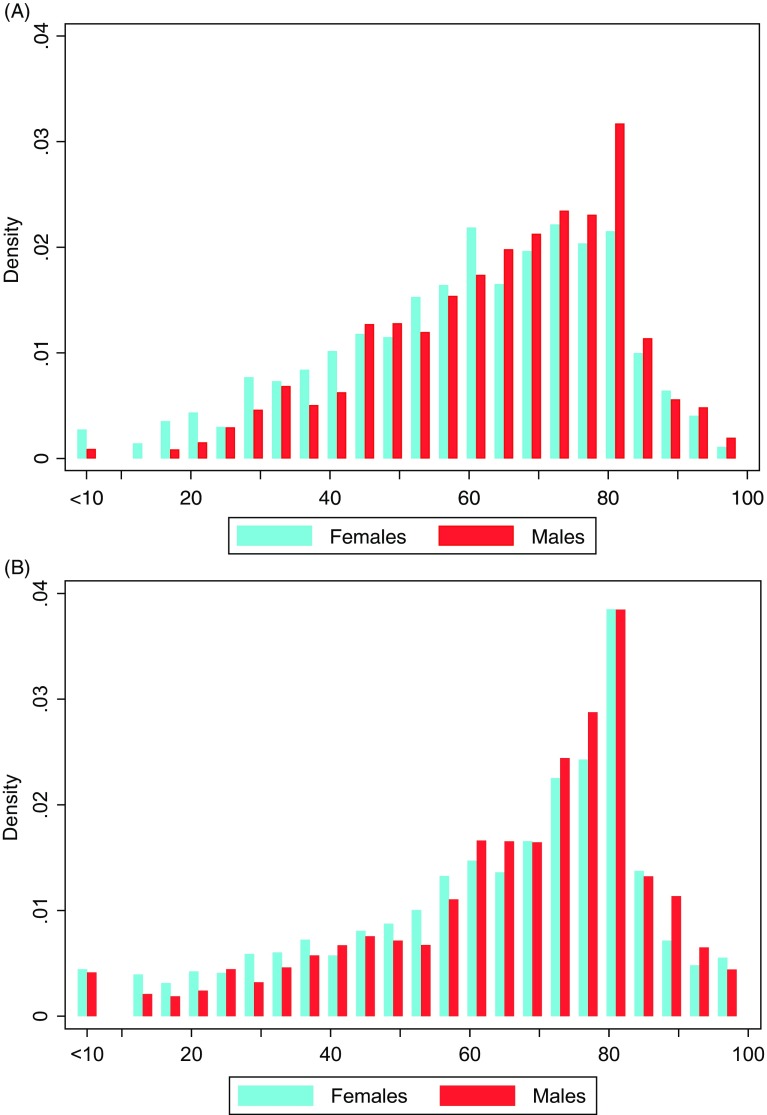
Histogram showing the distribution of WHO-5 scores stratified by gender. (A) The COVID-19 Consequences Denmark Panel Survey 2020 (*n* = 2458). (B) The Danish Mental Health and Well-Being Survey 2016 (*n* = 3501).

The mean age of respondents in the CCDPS 2020 was 49.1 years and 51% were females. The mean WHO-5 score was 62.0 for the total sample, 64.5 for males and 59.7 for females. The corresponding mean scores from the DMHWBS 2016 were significantly higher (64.3, *p* < 0.001; 65.8, *p* = 0.035; and 63.0, *p* < 0.001, respectively). Fig. 1 shows the distribution of WHO-5 scores by gender for the two surveys. The proportion of respondents from the CCDPS 2020 with WHO-5 scores <50 was significantly higher than for the DMHWBS 2016 survey for the total sample (25.4% vs. 22.5%, *p* < 0.001) and for females (28.8% vs. 24.6%, *p* = 0.005), but not for males (21.8% vs. 20.0%, *p* = 0.110). We found quite strong negative correlations between the reported levels of depression/anxiety and the WHO-5 scores (Table 1).

**Table 1.** Spearman correlation coefficients for the association between six self-reported symptoms of anxiety and depression (past 2 weeks) and the WHO-5 scores in the COVID-19 Consequences Denmark Panel Survey 2020

	Overall	Females	Males
Worry	−0.366	−0.385	−0.317
Nervousness	−0.525	−0.552	−0.472
Anxiety	−0.461	−0.464	−0.429
Depressed mood	−0.652	−0.657	−0.628
Hopelessness	−0.565	−0.575	−0.529
Guilt	−0.319	−0.314	−0.312

## Discussion

While we cannot rule out alternative explanations, the results of this study suggest that the psychological well-being of the general Danish population is affected negatively by the COVID-19 pandemic – and more so for females than for males. This resonates well with results from surveys conducted in other countries(American Psychiatric Association, 2020; Wang *et al.*, 2020; ) and will likely translate into increased demands for psychiatric treatment in the wake and aftermath of the pandemic.

## Supplementary material

For supplementary material accompanying this paper visit http://dx.doi.org/10.1017/neu.2020.15. click here to view supplementary material
