# Supplementary material for: The depressive state of Denmark during the COVID-19 pandemic
Source: Acta Neuropsychiatr. 2020 Apr 22:1–3. doi: 10.1017/neu.2020.15 (PMC7176490; doi:10.1017/neu.2020.15)
Supplement: Supplementary file 1 [file S0924270820000150sup001.docx]

**Supplementary Material**

**The COVID-19 pandemic and its practical consequences in Denmark:** The first confirmed case of COVID-19 in Denmark was reported by the State Serum Institute on February 26, 2020. Since then, there has been a steady increase in the number of reported cases and COVID-19-related deaths. During the fielding of the survey from March 31 to April 6, the average number of confirmed COVID-19 cases per day was 299 and the average number of COVID-19-related deaths per day was 15 (population of Denmark: 5.8 million).^1^ On March 11, the Danish government announced a nationwide lockdown involving closure of schools, kindergartens, teaching institutions, restaurants, bars and many small businesses. On March 23, the lockdown was extended to April 14 and was hence still in place during the fielding of the survey.

**The COVID-19 Consequences Denmark Panel Survey 2020:** In addition to the five-item WHO-5 well-being scale and the questions regarding the experienced level of anxiety and depression, the questionnaire included questions on a range of phenomena including social and institutional trust, political attitudes and values, and perception of the severity of the COVID-19 crisis. The survey was registered with the Danish Data Protection Agency via its agreement with Aarhus University. Ethical Review Board approval is not required for survey-based studies in Denmark.

**The Danish Mental Health and Well-Being Survey 2016:** The Danish Mental Health and Well-Being Survey 2016 (DMHWBS 2016)^2^ is a random representative sample of Danish men and women aged 16 years and above. Statistics Denmark sent an electronic letter to the sampled individuals in October 2016 with information about the study and an invitation to participate. After a week a reminder letter was sent, and after yet another week a final reminder was sent. A total of 1,656 men and 1,852 women (mean age = 47.0 years) responded to the web-based survey resulting in a response rate of 34%. The survey was registered with the Danish Data Protection Agency via its agreement with University of Southern Denmark.

**References**

1. Danish State Serum Institute webpage containing COVID-19 data from Denmark (<https://www.ssi.dk/aktuelt/sygdomsudbrud/coronavirus>). Accessed April 7, 2020.

2. Nielsen L, Hinrichsen C, Santini ZI, Koushede V. The Danish Mental Health and Well-Being Survey 2016. Statens Institut for Folkesundhed, Copenhagen, Denmark. 2017.
